# Supplementary material for: Net cost savings arising from patient completion of an active self-management program
Source: PLoS One. 2023 Nov 15;18(11):e0293352. doi: 10.1371/journal.pone.0293352 (PMC10650977; doi:10.1371/journal.pone.0293352)
Supplement: S1 Appendix — (DOCX) [file pone.0293352.s001.docx]

**S1 Appendix. Table 4.** Model parameters.

| Cost parameters | Description | Type of distribution | Mean | Lower 95% CI | Upper 95% CI | α | β | Source |
| --- | --- | --- | --- | --- | --- | --- | --- | --- |
| TKR | Cost of admission | γ | $ 22,385 | $15,530 | $ 29,546 | 81 | 275 | PHI |
| THR | Cost of admission | γ | $ 25,784 | $15,980 | $ 35,765 | 57 | 453 | PHI |
| Revision | Cost of admission | γ | $ 23,768 | $14,267 | $ 33,481 | 50 | 479 | PHI |
| Rehabilitation  (Inpatient) | Cost of admission | γ | $ 9,070 | $ 56 | $ 21,074 | 5 | 1,920 | PHI |
| Rehabilitation (RITH) | Cost of admission | γ | $ 2,817 | - | - | - | - | PHI |
| BKBM intervention | Cost of program |  | $ 2,246 | - | - | - | - | Harris et al. (2022) [1] |

| Secondary admissions | Description | Type of distribution | Estimate | Lower 95% CI | Upper 95% CI | α | β | Source |
| --- | --- | --- | --- | --- | --- | --- | --- | --- |
| TKR | Probability of admission | Point estimate | 0.20 | - | - | - | - | PHI |
| THR | Probability of admission | Point estimate | 0.05 | - | - | - | - | PHI |
| Revision | Probability of admission | Point estimate | 0.04 | - | - | - | - | PHI |
| Rehabilitation  (Inpatient) | Probability of admission | Point estimate | 0.55 | - | - | - | - | PHI |
| Rehabilitation (RITH) | Probability of admission | Point estimate | 0.45 | - | - | - | - | PHI |

|  |  | Treatment group | | | Control group | | |  |
| --- | --- | --- | --- | --- | --- | --- | --- | --- |
|  | Type of distribution | Mean | Standard error | 95% CI | Mean | Standard error | 95% CI | Source |
| **Baseline** |  |  |  |  |  |  |  |  |
| Willing | Truncated normal | 39% | 0.04 | [0.32, 0.46] | 39% | 0.06 | [0.27, 0.50] | Predicted |
| Unsure | Truncated normal | 35% | 0.04 | [0.28, 0.42] | 33% | 0.06 | [0.22, 0.44] | Predicted |
| Unwilling | Truncated normal | 26% | 0.03 | [0.20, 0.33] | 28% | 0.06 | [0.18, 0.39] | Predicted |
|  |  |  |  |  |  |  |  |  |
| **12 months post BKBM participation** |  |  |  |  |  |  |  |  |
|  |  |  |  |  |  |  |  |  |
| **Initially willing** |  |  |  |  |  |  |  |  |
| Remain willing | Truncated normal | 30% | 0.06 | [0.18, 0.41] | 56% | 0.12 | [0.33, 0.79] | Predicted |
| Willing to unsure | Truncated normal | 22% | 0.05 | [0.12, 0.32] | 22% | 0.10 | [0.03, 0.41] | Predicted |
| Willing to unwilling | Truncated normal | 48% | 0.06 | [0.36, 0.61] | 22% | 0.10 | [0.03, 0.41] | Predicted |
|  |  |  |  |  |  |  |  |  |
| **Initially unsure** |  |  |  |  |  |  |  |  |
| Unsure to willing | Truncated normal | 7% | 0.03 | [0.00, 0.13] | 33% | 0.11 | [0.12, 0.55] | Predicted |
| Remain unsure | Truncated normal | 24% | 0.06 | [0.13, 0.35] | 39% | 0.11 | [0.16, 0.61] | Predicted |
| Unsure to unwilling | Truncated normal | 69% | 0.06 | [0.57, 0.81] | 28% | 0.11 | [0.07, 0.48] | Predicted |
|  |  |  |  |  |  |  |  |  |
| **Initially unwilling** |  |  |  |  |  |  |  |  |
| Unwilling to willing | Truncated normal | 7% | 0.04 | [0.01, 0.14] | 14% | 0.09 | [0.10, 0.40] | Predicted |
| Unwilling to unsure | Truncated normal | 4% | 0.03 | [0.02, 0.10] | 7% | 0.07 | [0.10, 0.40] | Predicted |
| Remain unwilling | Truncated normal | 89% | 0.05 | [0.80, 0.98] | 79% | 0.11 | [0.57, 0.99] | Predicted |

**References**

1. Harris A, Hinman R, Lawford B, Egerton T, Keating C, Brown C et al. Cost effectiveness of telehealth-delivered exercise and dietary weight loss programs for knee osteoarthritis within a 12-month randomised trial. Arthritis Care & Research. 2022;.
