# Supplementary material for: Net cost savings arising from patient completion of an active self-management program
Source: PLoS One. 2023 Nov 15;18(11):e0293352. doi: 10.1371/journal.pone.0293352 (PMC10650977; doi:10.1371/journal.pone.0293352)
Supplement: S2 Appendix — (DOCX) [file pone.0293352.s002.docx]

**S2 Appendix. Table 5.** Cost and surgical rates for initial TKR assuming a probability rate of 0.65.

|  | Treatment group | | Control group | |
| --- | --- | --- | --- | --- |
|  | Surgeries | Cost | Surgeries | Cost |
| **12 months post BKBM participation** |  |  |  |  |
|  |  |  |  |  |
| **Initially unwilling** |  |  |  |  |
| Remain unwilling | 4 | $94,562.53 | 4 | $92,694.57 |
| Become willing | 7 | $141,843.87 | 16 | $337,071.05 |
| Become unsure | 0 | $9,456.24 | 1 | $16,853.56 |
|  |  |  |  |  |
| **Initially willing** |  |  |  |  |
| Remain willing | 52 | $1,105,759.74 | 94 | $1,976,409.01 |
| Become unwilling | 4 | $90,206.72 | 2 | $39,528.18 |
| Become unsure | 4 | $81,477.03 | 4 | $79,056.40 |
|  |  |  |  |  |
| **Initially unsure** |  |  |  |  |
| Becoming willing | 11 | $225,127.41 | 45 | $952,537.80 |
| Remain unsure | 4 | $78,794.61 | 2 | $39,689.07 |
| Become unwilling | 5 | $112,563.74 | 5 | $111,129.33 |
|  |  |  |  |  |
| Total | 92 | $ 1,939,791.9 | 173 | $ 3,644,969.0 |
| Per participant cost savings | $1,705.18 | | | |
