# Supplementary material for: Net cost savings arising from patient completion of an active self-management program
Source: PLoS One. 2023 Nov 15;18(11):e0293352. doi: 10.1371/journal.pone.0293352 (PMC10650977; doi:10.1371/journal.pone.0293352)
Supplement: S3 Appendix — (DOCX) [file pone.0293352.s003.docx]

**S2 Appendix. Table 6.** Cost and surgical rates for initial TKR assuming a probability rate of 0.65.

|  | Treatment group | | Control group | |
| --- | --- | --- | --- | --- |
|  | Secondary admissions | Cost | Secondary admissions | Cost |
| **12 months post BKBM participation** |  |  |  |  |
|  |  |  |  |  |
| Second TKR | 18 | $354,731.76 | 35 | $665,861.80 |
| THR | 5 | $102,150.56 | 9 | $191,745.32 |
| Revision | 4 | $75,329.29 | 7 | $141,399.51 |
| Rehabilitation (Inpatient) | 22 | $169,714.51 | 41 | $318,568.62 |
| Rehabilitation (RITH) | 18 | $43,268.96 | 33 | $81,219.53 |
|  |  |  |  |  |
| Total | 66 | $745,195.08 | 124 | $1,398,794.79 |
| Per participant cost savings from: |  | | | |
| Initial TKR | $1,705.18 | | | |
| Secondary admissions | $653.60 | | | |
| Cost of program | ($2,246) | | | |
| Net cost saving | $112.78 | | | |
