# Supplementary material for: Net cost savings arising from patient completion of an active self-management program
Source: PLoS One. 2023 Nov 15;18(11):e0293352. doi: 10.1371/journal.pone.0293352 (PMC10650977; doi:10.1371/journal.pone.0293352)
Supplement: Attachment — Submitted filename: Response to Reviewers.docx [file pone.0293352.s004.docx]

| **#** | **Journal feedback** | **Status** | **Author response** |
| --- | --- | --- | --- |
| 1 | The issues with this study are to do with the mix of input data and assumptions:  1) it begins with the premise that it will be a quantitative economic evaluation of observed data from an RCT, and thus have the rigour of causal inference on its side, but...; 2) it cannot due to a key unknown: the prior probability of a person progressing to surgery (over a given period) given the person's willingness status.  2) This means that the problem has both: 1) parameter uncertainty from observation error (i.e. the observed values of willingness are estimates from a sample of an underlying population, and thus are uncertain) as well as 2) assumption uncertainty (from the key unknown - assumed probability - forcing the analysts to test a range of plausible parameters).  And this shift from the initial premise to what was actually done makes the abstract very unclear for the reader. Expectations of a quantitative economic evaluation of observed data from an RCT are suddenly undermined in the space of a couple of sentences when suddenly "the analysis estimates the minimum probability required for the intervention to be..." - probability of what?? | Updated | Updated to include " The analysis estimates the minimum probability of progression to surgery required for the intervention to be cost-neutral when considering orthopaedic costs. " More broadly, we understand the reviewer's concern, but note that economic evaluations of RCTs can often require assumptions and modelling to address circumstances where long-term outcomes (in this case, if the participant goes on to have a joint replacement) have not yet been observed. We were left with wanting to highlight the economic implications of the RCT, while not observing key data. We chose a break-even analysis to explicitly acknowledge and highlight the assumption uncertainty that the reviewer mentions, and the impact it has on cost-effectiveness. |
| 3 | The reader has no idea yet what probability you're talking about. Nowhere in the abstract does this mysterious probability get revealed. The results report a proportion (60%) of initially willing participants proceeding to surgery. The study does not answer what proportion of initially willing participants actually did proceed to surgery, because it couldn't be observed in the trial beyond 1 year (or at all?) - has that actual number been reported anywhere? | Response provided | We have updated the abstract. Unfortunately due to ethical considerations, long term PHI data on TKRs post program was unavailable and the control group was not maintained.   This is stated in the Methods section. |
| 4 | The latter (2) makes this a Bayesian problem, in which the prior (2) has a strong influence on the results, with uncertainty (1) providing an uncertainty interval around all of the assumed probability input values. The methods do seem to capture all of this, but not very clearly. For example it is stated that "The probability of surgery given a participant's willingness status was treated as an uncertain parameter" (line 91) - I recommend you state up front in this paragraph that you were forced to make assumptions about this key unknown, and due to lack of good evidence were forced to model the full range of potential assumed values (from 0 to 1.0). | Updated | We have re-arranged the paragraph to start with the unknown component of our model, in line with the reviewer's comment. Additionally, to make things clearer, we have updated lines 100-102 to:  "A decision modelling framework that tested all probabilities of surgery given a participant’s stated willingness status in the future was used to conduct a break-even cost benefit analysis from the perspective of an Australian PHI. " |
| 5 | In the Discussion section, you list this as the second limitation - it should feature much more prominently in the Discussion than that. | Updated | We have brought forward as the major limitation as per reviewer comment |
|  | Then, be sure to distinguish this primary assumption from the secondary assumption (lines 100-3). | Updated | Updated wording slightly to " Given the lack of data on progression to surgery given a participant’s willingness status, we also assumed a fixed relationship between the probability of surgery given a participant’s willingness status to simplify the analysis. " (Lines 105-6) |
| 6 | I'm not clear where the standard errors in Table 3 come from. Are they from sampling uncertainty on the Likert scale of willingness? Table 5 makes me think that it is. I think Table 5 needs to come much earlier. | Response provided and updated | The standard errors come from sampling uncertainty.   Table 5 presents observed survey results prior to categorisation whereas Table 1 presents survey results post categorisation.   Table 5 has been moved to the methodology section and removed from appendix. |
| 7 | I think part of the lack of clarity is from the order we are provided the key information in. Also, it would be great to see a Figure or two with nice data visualisation, for example Table 1 could be presented visually showing what share of people shift from 'willing' to 'unsure' or 'unwilling', etc, over time. | Updated | Figure 2 showing proportion of "unwilling" for treatment and control groups has been included. |
| 8 | Table 5 is not consistent with Table 1: At baseline, Table 1 says that 39% of participants in both groups were "willing", but Table 5 says it was only 26% in the treatment group and 28% in the control group. This corresponds to the share that were "unwilling" in Table 1, so the classification labels may be transposed. Which is correct? This is a critical error, and could presumably make a big difference to the results. | Response provided | Apologies - there was an error in our original Table 5. Table 1 is correct and the former Table 5 has been updated. |
| 9 | Table 3 shows that the estimate for net savings becomes positive (cost saving) when the assumed probability of TKR surgery, given willingness, is 0.61 to 0.7. However the uncertainty interval based on the standard errors never excludes 0 even at the highest probability bracket. This is not entirely consistent with your Results and Conclusion in the abstract that claiming that "the intervention is cost saving", in terms of the uncertainty interval. | Updated | This is true however as per your later comment, there is a challenge between clarity of messaging and strictly statistical findings. We have tried to walk that balance - and have altered the text accordingly to be more succinct but less statistically definitive e.g. added "likely to be" cost saving. |
| 10 | I recommend stating very clearly in the abstract that the analysis is limited to the very narrow set of benefits (from lines 166-8). | Response provided | We have added text to the Abstract that notes we consider savings limited to reduced orthopaedic costs. |
| 11 | The Tables from the main paper seem to be repeated unnecessarily in the Appendices. | Updated | Removed. |
| 12 | Please address the assumption of constancy of the assumed probability over time (line 109) in the discussion. | Updated | Our study assumed a fixed annual transition probability for TKR over the 5-year horizon. Research into the progression to surgery suggests there are multiple pathways, some faster and some slower (Ref Törmälehto S, Aarnio E, Mononen ME, Arokoski JPA, Korhonen RK, Martikainen JA. Eight-year trajectories of changes in health-related quality of life in knee osteoarthritis: Data from the Osteoarthritis Initiative (OAI). PLoS One. 2019 Jul 19;14(7):e0219902. doi: 10.1371/journal.pone.0219902. PMID: 31323049; PMCID: PMC6641160.) so on balance we believe this is a reasonable assumption. However given cost savings are discounted, a higher likelihood of surgery in the first years relative to the later years would increase the pay-off to the intervention. |
|  | The interpretation is also too vague to make the study easily understandable and interpretable. In several place you state that "under certain conditions" and "in some circumstances..." (lines 159, 162, 187) the intervention may be "a dominant intervention", but don't clearly state what conditions, what circumstances? This should be made much clearer. | Updated | Updated to "Results from our orthopaedic cost model align with these studies, indicating that when at least 60% of willing participants go on to have surgery in the following 5 years, the intervention can deliver significant returns on investment to the PHI. "  Updated conclusion to "Results from our decision modelling framework indicate that at current rates of surgery such interventions can deliver significant returns on investment to the PHI." |
| 13 | The main driver appears to be (difference in) share of people who shift from willing or unsure to unwilling (according to Table 1, at least - but given inconsistency with Table 5 it's confusing). I suggest making this clearer in the discussion. | Updated | We have updated the tables and added text to highlight that the change in willingness is the key driver of the economic result. |
| 14 | The other main driver of course is the unknown key assumption. Give some clear guidance to help future researchers be very clear about what gaps in knowledge need to be addressed. | Reviewed | In the final para of the discussion we state " Additional research to better understand the occurrence of surgery given a participant’s willingness status and the possibility of perverse outcomes would assist with validating the robustness of extrapolating trial results to economic impact. " |
