# Supplementary material for: Net cost savings arising from patient completion of an active self-management program
Source: PLoS One. 2023 Nov 15;18(11):e0293352. doi: 10.1371/journal.pone.0293352 (PMC10650977; doi:10.1371/journal.pone.0293352)
Supplement: Attachment — Submitted filename: PlosOne - Feedback Tracker (22_Aug).docx [file pone.0293352.s005.docx]

| **Section/Area** | **Comment** | **Response** | **Status** |
| --- | --- | --- | --- |
| Methodology | Overall the framework is a sound one for answering this question, and the lack of observed surgery data is now clearer and well enough explained. Please specifically say whether it's a microsimulation model or what… | Thank you. The model is a decision analytic model. We have updated line 109 to state "Figure 1 below demonstrates the decision analytic modelling framework for the orthopaedic pathway. " It is also noted in the abstract (line 40). | Complete |
| Methodology | The sensitivity analyses use an ok approach. Much clearer now with the willingness status data in the tables and the modelling framework figure. | No action needed | Complete |
| Methodology | Clarify why only 1000 in the hypothetical cohort through the model (or is it 1000 in each of the intervention and control groups - it's ambiguous). | In line 109-111, we added 'each of' to clarify. "The model compares the occurrence and cost of TKR associated with a hypothetical cohort of 1000 participants in each of the treatment group and control group. " | Complete |
| Results | Line 149-150: an annual probability of 5.9% would result in only 27% conversion, not 60% - which would change the conclusions - please explain. | The 60% surgery rate is for willing participants. Only 39% of the total cohort are willing to have surgery. 34% are uncertain and 27% unwilling who have proportionally much lower probabilities of surgery. The sum product (39% X 60% + 34% X 6% + 27% X 3%) = 27% over 5 years or 5.9% each year. We have added text into the sentence in the results to clarify that the 60% only applies to those who are willing to have surgery - 39% of the total cohort. | Complete |
| Results | Table 4: the left column gives a range, but the other columns seem to come from a single value - clarify whether that single value the mid-point of the range, or are the values the average of model runs across all values in the range, or what specifically. - clarify whether the standard error and min-max range are due to parameter uncertainty or sampling variation between cohort runs, or both, or from sensitivity analyses. | Apologies - this was unclear. The results are from both varying the parameter within its 0.1 range and from varying the other input parameters as per Table 4 in the Appendix as part of a multivariate sensitivity analysis. We have revised the text and the table to clearly articulate this. | Complete |
| Discussion | The discussion states (line 166) that willingness "is the main driver" - may be overstating it as it was the only driver considered in this study. | We have revised to say that this explains the results, rather than this is the driver of the results. | Complete |
| Discussion | In the discussion, acknowledge the following as limitations: - assumption of the probability of surgery for unsure and unwilling to be 10% and 5% was fixed, and not varied in sensitivity analyses. | We have revised as requested and added "The unavailability of long-term surgical PHI data also meant our model assumed a fixed relationship between willingness states and progression to surgery and was not varied in our sensitivity analysis. " | Complete |
| Appendix | Appendix - table 4 line 312: why no uncertainty in the lower half of the table? these seem to be estimated from data. Appendix - table 4 line 313: These groups should not really differ at baseline in the model. Given people are randomly allocated to group, these are chance error only. The model should start both groups the same | The costs were provided as point estimates from the PHI and the program. The groups do not vary at baseline - both the treatment (BKBM group) and control have the same probability of attending inpatient rehabiliation (55%) or home-based rehabilitation (RITH - 45%). The table provides the probability of admission for secondary admissions after primary joint replacement. | Complete |
